# Reducing Defects in Organic-Lead Halide Perovskite Film by Delayed Thermal Annealing Combined with KI/I_2_ for Efficient Perovskite Solar Cells

**DOI:** 10.3390/nano11061607

**Published:** 2021-06-18

**Authors:** Kun-Mu Lee, Shun-Hsiang Chan, Wei-Hao Chiu, Seoungjun Ahn, Chang-Chieh Ting, Yin-Hsuan Chang, Vembu Suryanarayanan, Ming-Chung Wu, Ching-Yuan Liu

**Affiliations:** 1 Department of Chemical and Materials Engineering, Chang Gung University, Taoyuan 33302, Taiwan; shunhsiangchan@gmail.com (S.-H.C.); jun864@naver.com (S.A.); kero5206@gmail.com (C.-C.T.); cgu.yinhsuanchang@gmail.com (Y.-H.C.); 2 Division of Neonatology, Department of Pediatrics, Chang Gung Memorial Hospital, Linkou, Taoyuan 33305, Taiwan; 3 Green Technology Research Center, Chang Gung University, Taoyuan 33302, Taiwan; weihoa.chiu@gmail.com; 4 Center for Reliability Sciences and Technologies, Chang Gung University, Taoyuan 33302, Taiwan; 5 Electroorganic and Materials Electrochemistry Division, CSIR-Central Electrochemical Research Institute, Karaikudi 630003, India; vidhyasur@yahoo.co.in; 6 Department of Chemical and Materials Engineering, National Central University, Jhongli District, Taoyuan 32001, Taiwan

**Keywords:** perovskite solar cell, delayed annealing process, defect, additives

## Abstract

This study improved quality of CH_3_NH_3_PbI_3_ (MAPbI_3_) perovskite films by delaying thermal annealing in the spin coating process and introducing KI and I_2_ to prepare MAPbI_3_ films that were low in defects for high-efficiency perovskite solar cells. The influences of delayed thermal annealing time after coating the MAPbI_3_ perovskite layer on the crystallized perovskite, the morphology control of MAPbI_3_ films, and the photoelectric conversion efficiency of solar cells were investigated. The optimal delayed thermal annealing time was found to be 60 min at room temperature. The effect of KI/I_2_ additives on the growth of MAPbI_3_ films and the corresponding optimal delayed thermal annealing time were further investigated. The addition of KI/I_2_ can improve perovskite crystallinity, and the conductivity and carrier mobility of MAPbI_3_ films. Under optimized conditions, the photoelectric conversion efficiency of MAPbI_3_ perovskite solar cells can reach 19.36% under standard AM1.5G solar illumination of 100 mW/cm^2^.

## 1. Introduction

Perovskites, owing to their high absorption coefficient, long carrier diffusion length, high carrier mobility, low exciton binding energy, and controllable energy bandgap [1,2,3], have been extensively explored, especially those doped with organometallic halides (e.g., CH_3_NH_3_PbI_3_, MAPbI_3_). In recent years, the conversion efficiency of perovskite solar cells (PSCs) has increased from 3.8% [4] (as reported in 2009) to 25.5% [5] (in 2021). It is worth noting that the preparation of large-area PSCs with high efficiency and stability is critical for industrialization. The preparation of perovskites in accordance with the aforementioned conditions has been a research hotspot in recent years. Although the efficiency of PSCs has improved rapidly, there are still inevitable defects that affect the conversion efficiency and stability of solar cells. For example, the defect density (10^10^–10^11^ cm^−3^) of single-crystal MAPbI_3_ is lower than that of MAPbI_3_ (10^16^–10^17^ cm^−3^) prepared by solution deposition [6]. To our knowledge, the highest efficiency reported is 25.5%, which is still far from the theoretical maximum of 31% [7]. This can be attributed to the unavoidable shallow and deep defects generated during perovskite crystallization. This low efficiency may affect various aspects, including hysteresis [8], non-radiative recombination [9], carrier scattering [10], traps [11], and ion migration [12]. Therefore, it is critical to understand and control the defects in perovskite films.

For the crystal structure of perovskites such as ABX_3_, the cation in the A-site is of great importance to the photoelectric properties. Doped formamidine (FA) is commonly employed for A-site tuning, in which the FA and MA (methylammonium) are mixed in a prescribed ratio to efficiently increase the short-circuit current, owing to the radius of the FA ion being greater than that of the MA ion, and the fact that FA is quite stable in standard environments [13]. In this study, A-site tuning was also adopted by doping MAPbI_3_ with alkali metal cations. Note that alkali metal cations have smaller ion radii than that of MA, similarly to the relationship between MA and FA—the potassium ion, one of the alkali metals, has an ideal ion radius (1.38 Å). The potassium ion occupies interstitial vacancies in perovskite, and simultaneously generates MA vacancies (MA rotation causes potassium ions to enter) and prevents iodine ions from forming Frenkel defects. Therefore, the defects can be improved by doping with an appropriate amount of potassium ions, leading to improved crystallinity, a red shift of the optical energy band, and the ability to dissociate electron–hole pairs in perovskite films. Besides, it has been pointed out in previous studies that excessive doping will generate non-optical phase KPbI_3_ [14,15,16,17], facilitating precise control required for the doping.

In addition, theoretical calculations reported in previous studies indicate that point defects, including vacancies (V_MA_, V_Pb_, V_I_) and interstitial defects (MA_I_, Pb_I_, I_I_) [18,19], are easy to generate owing to the low formation energy of MAPbI_3_. Therefore, Yang et al. employed isopropanol to react with iodine to generate triiodide anion (I_3_^−^) to place FAPbI_3_ in an environment rich in polyiodide ions and avoid the generation of deep defects of FA. The repair of the aforementioned deep defects has been demonstrated via deep level transient spectroscopy (DLTS), and PSCs with efficiencies as high as 22.1% were fabricated [20]. Liu et al. performed DFT (density functional theory) calculations to study the point defects within FAPbI_3_, whose defect energy level is similar to that of MAPbI_3_ [21]. Therefore, in this study, based on the results reported previously, MAPbI_3_ films doped with mixed cations (e.g., K^+^ mixed with MA^+^) were further investigated. In addition, the triiodide anion was generated in well-controlled amount via a reaction between KI and I_2_ (I_2_ + I^−^ ⇄ I_3_^−^) by direct addition, which was similar to what took place in a previous study [22]. The I_3_^−^ generated within perovskite MAPbI_3_ was observed, and its ability to repair deep defects was studied. After a series of detection steps and analysis, the control over the preparation of perovskite films and the correlations between perovskite film defects controlled by additives and PSCs photovoltaic characteristics were explored.

## 2. Materials and Methods

A 30 nm-thick TiO_2_ compact layer (c-TiO_2_) was deposited on top of the FTO glass substrate by spraying a solution of titanium diisopropoxide bis(acetylacetonate) (75 wt.% of Ti(acac)_2_OiPr_2_ in isopropanol, Sigma-Aldrich, Burlington, VT, USA) at 450 °C. A 150 nm-thick mesoporous TiO_2_ (m-TiO_2_) thin film, which had an average particle size of ~20 nm, was printed on top of c-TiO_2_/FTO glass substrate using a home-made paste and heated to 500 °C for 30 min. After being cooled to room temperature, the samples were transferred to a nitrogen-filled glove box (<5%RH). The perovskite precursor consisted of PbI_2_ (1.8 M, 99.9985%, Alfa Aesar, Haverhill, MA, USA) and CH_3_NH_3_I (1.8 M, >98%, STAREK^®^) in DMSO (99.9%, ECHO, Miaoli, Taiwan) and γ-butyrolactone (GBL, ≥99%, across, Livingston, NJ, USA) (5/5, *v*/*v*); potassium iodide (KI, >99%, acros) dissolved in DMSO/GBL (5/5, *v*/*v*) was added to perovskite precursors in various molar ratios (0.01, 0.03, or 0.05 M). In a similar process, the iodine (I_2,_ <99.8%, Sigma-Aldrich, Burlington, VT, USA)) was added to perovskite precursors in various molar ratios (1.25, 2, 3, or 6 mM), respectively. The perovskite precursor was spin-coated on top of the m-TiO_2_/c-TiO_2_/FTO glass substrate with an anti-solvent washing enhanced nucleation process [20,21,23,24]. The perovskite films were allowed to stand for various amounts of time (0, 30, 60, 90, and 120 min) at room temperature in glass culture dishes before thermal baking at 100 °C for 10 min; the time before baking is called “delayed thermal annealing time”. Spiro-OMeTAD (99.93%, Ruilong, Miaoli, Taiwan) was dissolved in chlorobenzene (50 mg/mL), and 17.5 μL of a solution of lithium bis(trifluoromethane)sulfonimide (Li-TFSI, 520 mg, >98%, Alfa Aesar, Tewksbury, MA, USA) in acetonitrile (1 mL) and 28.5 μL 4-tert-butylpyridine (96%, Alfa Aesar) were added to the Spiro-OMeTAD solution. Then, the solutions were stirred and heated to 60 °C for 2 h. Spiro-OMeTAD solution was spin-coated on top of perovskite/m-TiO_2_/c-TiO_2_/FTO glass substrate at 2000 rpm for 30 s. Finally, a 100 nm-thick Ag film was deposited by the thermal evaporation method as the top electrode. The active area of PSC was fixed at 0.16 cm^2^ (and 1.21 cm^2^ for comparison). The AM 1.5G solar simulator (SS-F5-3A, ENLI Technology Co. Ltd., Kaohsiung, Taiwan) was used as the irradiation light source for the current density–voltage (J–V) measurements. The illumination intensity of 100 mW/cm^2^ was calibrated before cell performance measurement. The J–V characteristics of the PSCs under the illumination of AM 1.5G simulated sunlight were obtained by applying the external potential bias to the cell and measuring the photocurrent output with a Keithley model 2400 digital source meter (Keithley, Beaverton, OR, USA). The incident photon conversion efficiency (IPCE) spectrometer (QE-R-3011, ENLI Technology Co. Ltd., Taiwan) calibrated with a single-crystalline silicon reference cell was used for the IPCE measurements. The photoluminescence (PL) spectra and time-resolved PL spectra were recorded with a 532 nm diode laser (LDH-D-TA-530, PicoQuant, Berlin, Germany). The temperature-dependent PL spectra were recorded using Linkam THMS600 stage. The TRPL plots were recorded by a time-correlated single-photon counting (TCSPC) (TimeHarp 260, PicoQuant) spectrometer. Surface morphologies of samples were recorded by a tapping-mode atomic-force microscope (AFM) (Nano-Scope NS3A system, Digital Instruments, Bresso, Italy) and a field emission scanning electron microscope (SEM) (SIGMA, Zeiss, Jena, Germany). Each XRD spectrum was obtained using an X-ray diffractometer (D8 130 Focus, Bruker, Dresden, Germany). UV–Vis spectra of samples were measured by a broadband spectrometer (U-4100, HITACHI, Tokyo, Japan).

## 3. Results and Discussion

### 3.1. The Effect of Delayed Thermal Annealing on the Morphological Characteristics of the Surfaces of MAPbI_3_ Perovskite Films

**Figure 1:**
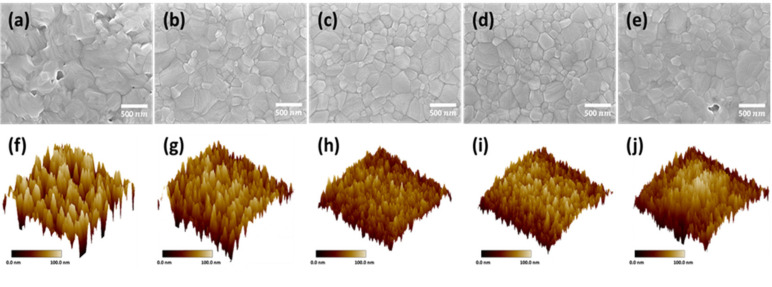
SEM and AFM of perovskite films’ surface structures. The films were prepared with different delay-annealing times: (**a**,**f**) 0 min; (**b**,**g**) 30 min; (**c**,**h**) 60 min; (**d**,**i**) 90 min; (**e**,**j**) 120 min.

The surface morphology of the MAPbI_3_ perovskite films under different delayed thermal annealing times were investigated, and the images are reported in Figure 1. Figure 1a,f shows the films after thermal annealing at 100 °C, immediately after spin coating (without standing time), which can reveal the influences of the Marangoni effect on perovskite film formation. In this study, GBL and DMSO were mixed in a volume ratio of 1:1 to form a perovskite precursor solution. Due to the strong surface tension and viscosity possessed by DMSO, a surface tension gradient drop occurs during perovskite film formation [23,24], leading to an uneven crystallization rate and excessive crystal grains. The speed in the vertical crystallization direction is greater than that in the horizontal crystallization direction, resulting in poor uniformity of the film. It is easy for many island-shaped crystal forms to cause uneven crystal grain size and higher roughness, resulting in a low coverage film. This leads to a poor contact between the perovskite and electron/hole transport layer, and the electron/hole conductive layers cannot be effectively isolated. Figure 1b–e,g–j shows various delayed thermal annealing conditions. In comparison to the process without delay, a delayed treatment procedure resulted in better crystal morphology.

Under the delayed thermal annealing process, the uneven crystallization rate caused by surface gradient tension is balanced and the crystals are closely arranged; the grain boundaries are obvious. The disappearance of holes in a perovskite film improves the film coverage, which is helpful for electron/hole division and transportation. This can be verified from the maximum roughness (R_max_) and average roughness (RMS) of AFM analysis. When the delayed thermal annealing time was 60 min, the Rms decreased significantly—from 56 nm without the delay process to 19 nm. However, when the delayed thermal annealing time was prolonged for more than 60 min, the Rms increased again, and the relevant values are listed in Table 1.

**Figure 2:**
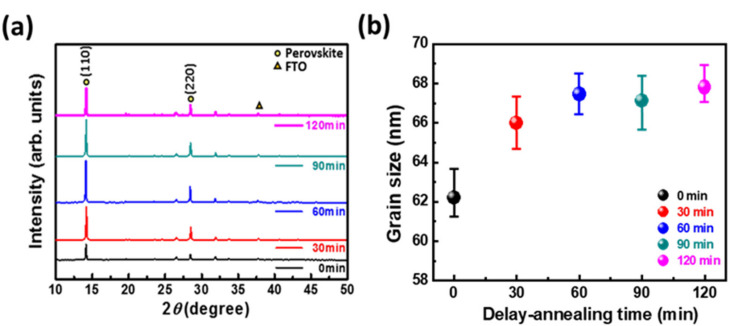
(**a**) XRD of perovskite films prepared with various delay-annealing times. (**b**) Crystallite size of perovskite films.

Two reasons can be speculatively presented for this phenomenon. The first is that gravity counteracts the cohesion created by DMSO, which causes the holes to be generated again. The second is that the perovskite films are not well aligned during the annealing process. Similar results can be obtained from AFM and SEM analysis. By prolonging the delayed thermal annealing time, the uneven crystallization rate of perovskites can be modified and the scale of crystallization in the vertical direction can be reduced. The flatness and coverage of perovskite films can obviously be improved. Therefore, the optimal experimental condition for the delayed annealing time was 60 min. For the crystal phase analysis of perovskite films, XRD was used to observe the change of perovskite crystallization caused by the delayed annealing process. As shown in Figure 2a, 14.1° and 28.4° were the two main diffraction peaks of the perovskite tetragonal crystal structure, corresponding to the crystal lattice planes of (110) and (220), respectively. The figure shows that as the delayed thermal annealing time increased from 0 to 60 min, the perovskite crystallinity gradually increased. It is speculated that in delayed thermal annealing, a slower crystallization rate makes the structure bonds complete, and the crystallinity and stability of the perovskite film are improved. However, when the delayed thermal annealing time exceeded 60 min, the crystallinity of the perovskite began to decrease, due to the aggregation of the perovskite precursor. The full width at half maximum values of the main diffraction peaks of the perovskite (14.1°) in the XRD spectrum were calculated (0.130, 0.123, 0.119, 0.121, and 0.118 for 0, 30, 60, 90, and 120 min, respectively), and the sizes of the crystals of perovskite under various conditions were measured, as shown in Figure 2b. The crystal size slightly increased from 62 nm without treatment to 68 nm after a delayed thermal annealing time of 60 min because this process provides an equilibrium time for crystal growth, in which the crystals can fuse with each other, resulting in more uniform and larger crystals.

**Figure 3:**
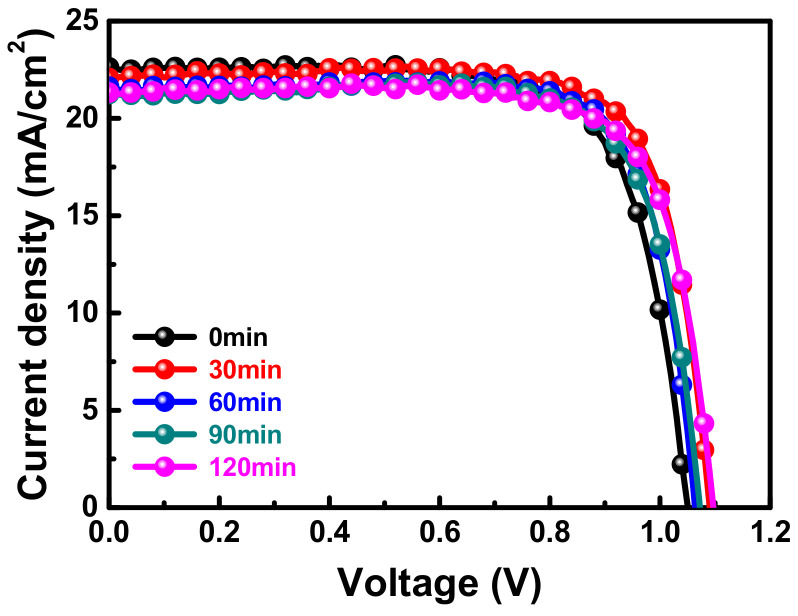
J–V curves of PSCs prepared with various delayed annealing times.

Figure 3 shows the J–V plot of PSCs with different delayed thermal annealing times, and the relevant photovoltaic parameters are listed in Table 2. The open-circuit voltage (V_OC_) of the PSC increased to approximately 1.07 V after delayed thermal annealing treatment. It was also found that the delayed thermal annealing process can rearrange crystals of perovskite film and make the surface smoother. Moreover, the electron transport layer/perovskite and/or hole transport layer/perovskite showed fine contact due to the smooth perovskite film, leading to smaller internal series resistance of the cell. The fill factor (FF) increased to 78% and the optimal delayed thermal annealing time was 60 min. Therefore, the 60 min thermal annealing time is adopted for further discussion.

### 3.2. Reducing Defects in Perovskite Films by Doping with Potassium Iodide and Iodine

**Figure 4:**
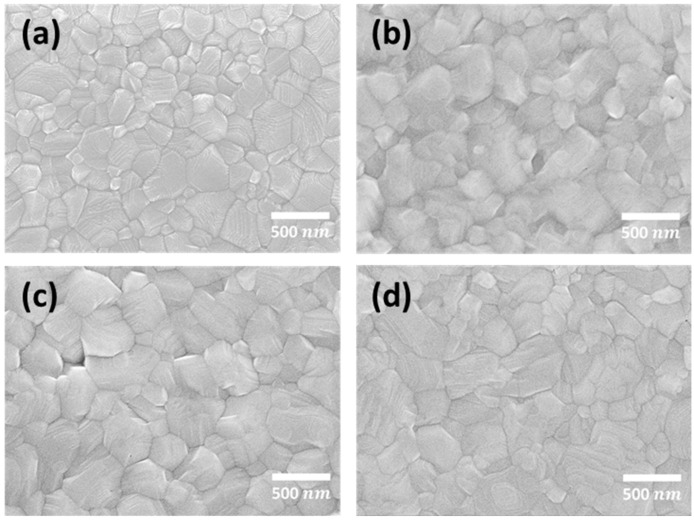
SEM top view of (**a**) perovskite film without KI (thermal baking time: 10 min); and perovskite films with 0.03 M KI under various thermal baking times: (**b**) 10 min, (**c**) 30 min, and (**d**) 50 min.

The preparation of perovskite thin films inevitably generates defects, which can be minimized by the introduction of different alkali metal halides, as revealed by previous studies [25,26]. Specifically, as an additive, KI exhibits the best performance owing to the appropriate size of the cation provided. Therefore, the doped potassium ions were adopted to generate interstitial defects to avoid the generation of Frenkel defects by iodine ions, which is an approach to improve defects by manipulation. The optimal delayed thermal annealing time was 60 min, as revealed in the previous studies, and KI was first added for doping. Subsequently, I_2_ was added to generate I_3_^−^ (I_2_ + I^−^ → I_3_^−^) to repair the deep defects in perovskite films and improve the quality of the films. As revealed by previous studies [27,28,29,30,31] on the preparation of perovskite with mixed cations or mixed anions, prolonged baking times and high baking temperatures after film preparation are the most common methods adopted in the film crystallization process. The crystallization rate decreases after the introduction of KI, and therefore, the optimal thermal baking time needed to be investigated. Different prolonged thermal baking times (i.e., 10, 30, and 50 min) were compared for perovskite films doped with KI (30 mM). Figure S1 shows the J–V plot of perovskite films with different thermal baking times. The relevant photovoltaic characteristic data are given in Table S1. The prolonged thermal baking time indeed helped perovskite crystallization to complete, thereby efficiently increasing the value of FF. For a thermal baking time of 30 min, better V_OC_ (1.086 V), FF (75.1%) and conversion efficiency (18.93%) were obtained. Moreover, from AFM analysis (as shown in Figure S2), the average roughness of the perovskite film decreased with delayed thermal annealing time (as shown in Table S2), indicating that the delayed thermal annealing process still leads to consistent results after KI doping. As seen in SEM analysis (as shown in Figure 4), after the perovskite film doped with KI was baked for 10 min, there were many small grains at the boundaries of the large grains, indicating that the energy provided by brief baking is not sufficient to complete the crystallinity. The issue of incomplete crystallization was addressed if the thermal baking time was prolonged to 30–50 min, in which case large grains and grain boundaries were obvious. Figure 4a shows that in comparison with perovskite films without KI doping, the those with such doping had larger grains on average and fewer grain boundaries, which reduced the possibility of defect distribution in the films and the obstruction of charge transfer due to the existence of the grain boundaries.

Furthermore, XRD was used to analyze the effect of baking time on the crystallization of perovskite films after doping with KI (as shown in Figure S3). After 30 min, the crystallinity of the perovskite was improved, indicating that it takes more baking to complete the crystallization after the introduction of KI. A similar result was obtained when the baking time was prolonged to 50 min, with crystal sizes of approximately 68–70 nm, which is consistent with the SEM results. Furthermore, photoluminescence (PL) and time-resolved photoluminescence (TRPL) were used to investigate the photophysical behavior of perovskites doped with KI and baked for various amounts of time, during which the dissociation and transfer behavior of carriers in perovskite films and from the perovskite to the electron transport layer were measured. The perovskite film was deposited on the electron transport layer of TiO_2_/FTO glass. Figure S4a shows the PL results, revealing that when the baking time was 10 min, the fluorescence intensity was the highest, demonstrating that the crystallization was incomplete, which is consistent with SEM results. The reason behind this phenomenon is that after light excitation, it is impossible to dissociate sufficient electrons and holes that reach the carrier transport layer, resulting in a recombination reaction inside the perovskite and production of much fluorescence. After baking for 30 min, the perovskite crystal growth was complete, and recombination was significantly suppressed, leading to low photoluminescence intensity. This effect occurred for both situations when the baking time was extended to 30 or 50 min—they presented similar trends, as revealed by SEM and XRD analysis results, validating each other. Figure S4b shows the TRPL results, along with the average carrier lifetime calculated by formula fitting, as given in Table S3. In particular, the optimal baking time was 30 min, with the lowest average carrier lifetime being 10.2 ns, which indicates the excellent electron and/or hole dissociation ability after thermal treatment for 30 min.

As pointed out in previous studies, I_3_^−^ can reduce the deep defects in perovskites and effectively improve the efficiency of PSC. Therefore, a method different from those reported in previous studies [20,32] which produces I_3_^−^ by reacting iodine with isopropyl alcohol in a time-consuming manner (~7 days) was adopted. Specifically, the optimal KI doping condition described previously was employed to directly add a small amount of iodine (i.e., 1.5, 2, 3, or 6 mM) to react with I^−^ existing in the solution to spontaneously generate I_3_^−^ in equilibrium (I^−^+ I_2_ ⇄ I_3_^−^, pK = 10^7^ L mol^−1^). This method was expected to simultaneously retain the advantages of potassium ions and triiodide anions.

As the delayed thermal annealing process is critical to the treatment procedure, the AFM analysis was also used to understand the influence of KI/I_2_ doping in perovskite films, as shown in Figure S5 and Table S4. After the delayed thermal annealing, the film surface roughness still decreased. The maximum roughness height (R_max_) was exhibited after 60 min under the optimal conditions, which was approximately 100 nm away from that of the control one. Besides, RMS was reduced from 33.5 to 26.2 nm, but the effect was not significant compared with that in the previous treatment, indicating that the addition of KI/I_2_ has the effect of reducing the roughness of perovskite films and making the delayed thermal annealing less effective.

The J–V plot of the PSC component with different KI/I_2_ concentration ratios is shown in Figure S6. The photovoltaic results are listed in Table 3. In the presence of I_3_^−^, the V_OC_ and FF of the PSCs considerably increased, which could have been related to the repair of deep defects in the material. Specifically, optimal PSC performance was obtained when the ratio of KI/I_2_ was 10/1 (iodine concentration set as 3 mM), including the highest PCE of 19.36% (active area = 0.16 cm^2^), a V_OC_ of 1.091 V, a J_SC_ of 22.83 mA cm^−2^, and an FF of 77.7%. Furthermore, it also achieved a nice PCE for a larger active area (1.21 cm^2^) of 16.50%.

**Figure 5:**
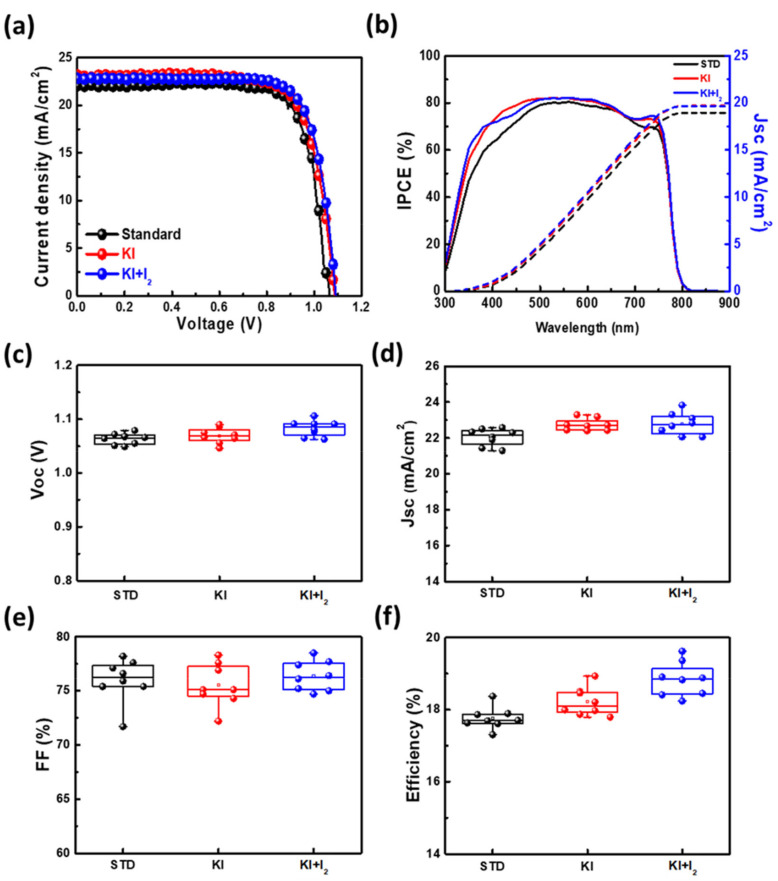
(**a**) J–V curves, (**b**) IPCEs, and (**c**–**f**) the statistical photovoltaic characteristics of PSCs with various optimal conditions (pristine, KI (30 mM), and KI (30 mM) + I_2_ (3 mM)).

**Figure 6:**
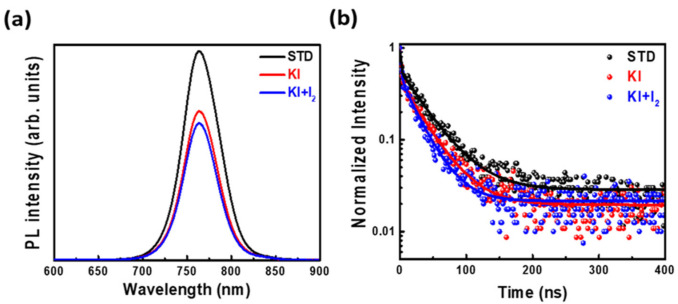
The (**a**) PL and (**b**) TRPL analyses of perovskite films on TiO_2_/FTO glass with various optimal conditions (STD, KI (30 mM), and KI (30 mM) + I_2_ (3 mM)).

**Figure 7:**
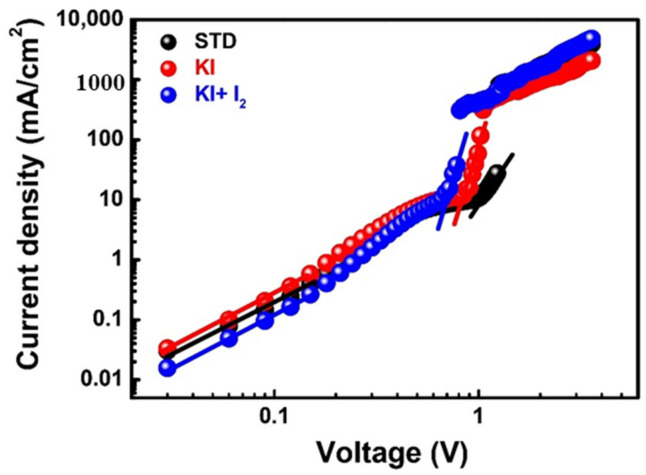
The SCLC measurements of perovskites films with various optimal conditions (STD, KI (30 mM), and KI (30 mM) + I_2_ (3 mM)).

For further investigation, pristine perovskite (STD), perovskite with doped KI, and KI/I_2_ were compared in terms of photovoltaic performance of the PSCs and photoelectric properties of perovskite films. The optimal efficiency J–V curves, IPCEs, and statistical photovoltaic characteristics of PSCs for various conditions are shown in Figure 5. The results show that V_OC_ and FF substantially increased, which may have been due to the reduction of Frenkel defects, the repair of deep defects, and less carrier migration hindrance between the perovskite layer and the electron/hole transport layer. Meanwhile, the J–V curves with the hysteresis index of pristine and KI/I_2_ samples are shown in the Supporting Information (Figure S7). It was found that PSC with KI/I_2_ had a lower HI value (~0.033) than the pristine one (~0.068), which could support the perovskite film with KI/I_2_ showing lower defects, and this can be mutually confirmed with analysis of results in Figure 6 and Figure 7 below. Furthermore, a better IPCE value was obtained either in the short-wavelength range or the long-wavelength range (Figure 5b), which means that the perovskite crystal structure and internal defects of the material can be improved after the treatment and indeed have the better statistical photovoltaics characteristics (Figure 5c–f).

In addition, the perovskite films under the above three conditions were analyzed using PL and TRPL. Figure 6a shows that undoped perovskite film had the strongest photoluminescence intensity, which may have been contributed by the presence of many traps in perovskite films, resulting in electrons and holes inside the perovskite film recombining. In contrast, for doped perovskite films, electrons are dissociated and transported to the electron transport layer, resulting in less recombination and lower PL intensity. In addition, the TRPL analysis of carrier lifetime trends is shown in Figure 6b and Table 4. The TRPL spectra was fitted to a bi-exponential decay function:

$$F\left(t\right)=A_{1}\exp\left(-\frac{t}{\tau_{1}}\right)+A_{2}\exp\left(-\frac{t}{\tau_{2}}\right)$$

where $\tau_{1}$ and $\tau_{2}$ are fast decay lifetime and slow decay lifetime, respectively. $A_{1}$ and $A_{2}$ are the corresponding amplitudes. The average carrier lifetime ($\tau_{\mathrm{avg}}$) was estimated using the following equation:

$$\tau_{\mathrm{avg}}=\left(A_{1}\tau_{1}+A_{2}\tau_{2}\right)/\left(A_{1}+A_{2}\right)$$

The results reveal that perovskite films doped with KI/I_2_ had the shortest $\tau_{\mathrm{avg}}$, leading to the reduction of defects and promotion of the charge separation.

To further analyze the electrical properties of perovskite films under different doping conditions, space charge-limited current (SCLC) analysis was performed, as shown in Figure 7, and data are listed in Table 5. We fabricated the electron-only devices that consisted of FTO/c-TiO_2_/m-TiO_2_/perovskite/PCBM/Ag. Those curves can be divided into three sections for comparison. The first section shows the conductivity of the material following Ohm’s law, which exhibits negligible variations for perovskite films with or without KI doping. In contrast, after the introduction of KI/I_2_ to generate I_3_^−^, the conductivity of perovskite film increased by approximately three times (from 4.42 × 10^−4^ to 1.22 × 10^−3^ mS cm^−1^), owing to the repair of deep defects. Seto et al. also pointed out that deep defects in perovskite grain boundaries can hinder carriers and reduce carrier mobility and conductivity [33]. In the second section, the relevant slope of the curve increases sharply, referred to as the TFL (trap filled limit) region. Note that the trap-filling behavior of carriers can be revealed by the analysis of the defect density, which was 1.09 × 10^16^ cm^−3^ for pristine perovskite and was reduced to 8.22 × 10^15^ cm^−3^ after doping with KI, and further to 6. 63 × 10^15^ cm^−3^ after doping with KI/I_2_ (I_3_^−^). The decrease in defect density validates the fact that after doping with KI and KI/I_2_, the quality of perovskite films can be improved (lower defect density). The third section is one with space charge-limited current, in which carrier mobility was investigated, to obtain the carrier migration behavior. After doping with KI, carrier mobility little decreased from 8.40 × 10^−2^ to 3.52 × 10^−2^ cm^2^ V^−1^ s^−1^. When I_3_^−^ was formed after doping with KI/I_2_, the carrier mobility increased to 1.26 × 10^−1^ cm^2^ V^−1^ s^−1^, and this confirmed that the improvement in the deep defects of perovskite films can improve the conductivity and carrier mobility, thereby improving the photovoltaic performance of PSCs.

**Figure 8:**
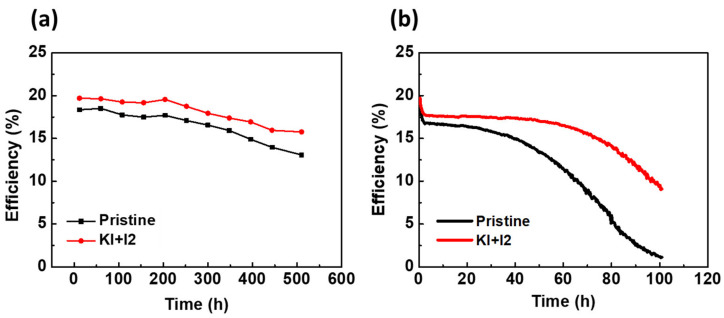
The stability of PSCs without encapsulation at 25 °C and ~35 RH%: (**a**) during at-rest stability (dark storage) and (**b**) during continuous light soaking under 1 sun.

For investigating the stability of PSC, dark storage study provides information on the tolerance of the solar cells to oxygen, moisture, and other aggressive atmospheric components naturally present in air. It estimates a cell’s shelf life under ambient conditions when it is not exposed to light. On the other hand, a light soaking test promotes ion and defect migration in PSCs, along with phase segregation in the perovskite layer, which reduces conversion efficiency. Acceleration of harmful chemical reactions leading to either perovskite decomposition or defect formation can also be caused by device illumination [34,35,36]. Figure 8a shows the dark storage of PSC with KI/I_2_, demonstrating a stability improvement after being exposed to an ambient atmosphere (∼35% relative humidity, 25 °C) for more than 500 h without encapsulation. The pristine PSC and PSC with KI/I_2_ reached 71.2% and 80.1% of their initial PCEs after 500 h, respectively. This indicates that the perovskite films with KI/I_2_ having lower defect density and better carrier mobility, and a low hysteresis index (shown in Figure S7), could improve the at-rest stability of perovskite solar cells, which could be consistent with the previous literature [37,38]. Figure 8b shows the continuous light soaking of PSCs under illumination of AM 1.5, 1 sun. The PSC with optimized KI/I_2_ had a lower defect density, providing a better stability with a small drop in which it underwent the first reversible transformation (formation of light-activated trap states or photo-induced halide ion segregation) [36,39]. However, the efficiency significantly declined with extended continuous light soaking time, leading to some irreversible degradation or defect formation in perovskite film or interfaces [40]. Li et al. also pointed out the role of iodide vacancies as the main migrating species with respect to iodide ions. The light-induced electrical field in the film can cause the iodide ions/vacancies to migrate [41]. The addition of KI/I_2_ in perovskite can effectively reduce this phenomenon, which is in consistent with the results shown in Figure 6 and Figure 7 of this study.

## 4. Conclusions

In this study, a series of perovskite film formation control methods involving additives were adopted to successfully improve the conversion efficiency of perovskite solar cells. We introduced a delayed thermal annealing process to balance the influence of the Marangoni effect on the perovskite film crystallization, resulting in decreased film surface roughness and increased crystallization strength; these effects can be also maintained in subsequent doping process. Potassium iodide and iodine doping were successively conducted to reduce the defects in perovskite films. Doping with potassium ions and the generation of new defects (V_MA_) prevented the formation of Frenkel defects by iodide ions, reducing the overall defect density, thereby improving the crystallization of perovskite. Finally, the reaction between iodide ions and iodine was utilized to partly obtain the triiodide anions, which further improved film conductivity and carrier mobility in perovskite films, resulting in excellent PSCs performances, as demonstrated by the power conversion efficiency of 19.36%. The at-rest stability and continuous light soaking tests of PSCs were also carried out under 25 °C and ~35 RH%.

## Figures and Tables

**Table 1:** The R_max_ and Rms roughness of perovskite films prepared with various delayed annealing times.

Time (min)	0	30	60	90	120
R_max_ (nm)	398	268	154	137	241
RMS (nm)	56	33	19	21	32

**Table 2:** The photovoltaic characteristics of PSCs with perovskite films prepared with various delayed annealing times.

Time (min)		V_OC_ (V)	J_SC_ (mA cm^−2^)	FF (%)	PCE (%)	Rs (ohm)
0	Maximum	1.050	22.62	73.4	17.45	9.2
	Mean deviation	1.046 ± 0.010	22.19 ± 0.45	73.3 ± 0.6	17.05 ± 0.61	-
30	Maximum	1.075	22.28	77.0	18.44	6.6
	Mean deviation	1.076 ± 0.003	22.06 ± 0.57	74.2 ± 0.4	17.61 ± 0.76	-
60	Maximum	1.072	22.05	78.2	18.48	5.2
	Mean deviation	1.074 ± 0.011	22.04 ± 0.47	76.9 ± 0.1	18.20 ± 0.31	-
90	Maximum	1.072	21.24	76.9	17.53	6.7
	Mean deviation	1.076 ± 0.005	20.71 ± 0.76	76.6 ± 0.3	17.10 ± 0.62	-
120	Maximum	1.096	21.32	76.3	17.85	6.9
	Mean deviation	1.082 ± 0.008	21.01 ± 0.36	75.7 ± 1.4	17.25 ± 0.60	-

**Table 3:** The photovoltaic characteristics of PSCs with various concentrations of I_2_.

Concentration (mM)		V_OC_ (V)	J_SC_ (mA cm^−2^)	FF (%)	PCE (%)
1.5	Maximum	1.101	23.46	70.7	18.26
	Mean deviation	1.090 ± 0.014	23.13 ± 0.27	71.0 ± 1.6	17.92 ± 0.32
2.0	Maximum	1.102	22.99	73.5	18.60
	Mean deviation	1.090 ± 0.008	23.15 ± 0.42	72.0 ± 1.4	18.17 ± 0.55
3.0	Maximum ^a^	1.091	22.83	77.7	19.36
	Mean deviation ^a^	1.083 ± 0.014	22.78 ± 0.61	76.3 ± 1.4	18.83 ± 0.47
	Maximum ^b^	1.085	22.22	68.44	16.50
	Mean deviation ^b^	1.079 ± 0.016	22.05 ± 0.72	67.2 ± 1.6	15.99 ± 0.66
6.0	Maximum	1.074	22.18	72.7	17.33
	Mean deviation	1.080 ± 0.021	21.89 ± 0.36	72.0 ± 2.4	17.10 ± 0.34

^a^ active area = 0.16 cm^2^; ^b^ active area = 1.21 cm^2^.

**Table 4:** List of the fast decay lifetime (τ_1_), slow decay lifetime (τ_2_), and PL average decay (τ_avg_) of the TiO_2_/FTO glass with various perovskite films.

Additive	A_1_ (%)	τ_1_ (ns)	A_2_ (%)	τ_2_ (ns)	τ_avg_ (ns)
STD	40	1.3	60	38.4	23.5
KI (30 mM)	55	1.7	45	34.3	16.4
KI + I2 (30 mM + 3 mM)	73	1.9	27	44.1	13.3

**Table 5:** The SCLC data of PSCs with various optimal conditions (STD, KI, and KI/I_2_).

Condition	Electrical Conductivity (mS cm^−1^)	V_TFL_ (V)	Trap Density (cm^−3^)	Mobility (cm^2^ V^−1^ s^−1^)
STD	4.42 × 10^−4^	1.23	1.09 × 10^16^	8.40 × 10^−2^
KI (30 mM)	4.48 × 10^−4^	0.93	8.22 × 10^15^	3.52 × 10^−2^
KI (30 mM)/I_2_ (3 mM)	1.22 × 10^−3^	0.75	6.63 × 10^15^	1.26 × 10^−1^

## Data Availability

The data presented in this study are available on request from the corresponding author.

## Supplementary Materials

The following are available online at https://www.mdpi.com/article/10.3390/nano11061607/s1, Figure S1: The J–V curves of PSCs with 30 mM KI under various thermal baking time, Figure S2: The AFM images of perovskite film with 30 mM KI at different delay-annealing times, Figure S3: (a) XRD analysis of perovskite films with 0.03 M KI under various thermal baking times. (b) Crystal size in perovskite films with 0.03 M KI under various thermal baking times, Figure S4: (a) PL and (b) TRPL of perovskite films with 0.03 M KI under various thermal baking times, Figure S5: AFM of perovskite films with 0.03 M KI/I2 at different delayed annealing times, Figure S6: The J–V curves of PSCs with 30 mM KI and various concentrations of I2, Figure S7: The J–V curves of PSCs by forward (Black) and reverse scan (Red). (a) Pristine and (b) KI (30 mM) + I2 (3 mM), Table S1: The Rmax and RMS of perovskite films with 30 mM KI prepared at different delayed annealing times, Table S2: Photovoltaic characteristics of PSCs with 30 mM KI at different thermal baking times, Table S3: The carrier lifetimes of perovskite with 30 mM KI fitting from TRPL analysis at different thermal baking times, Table S4: R_max_ and RMS of perovskite films with KI (30 mM)/I_2_ (3 mM) at various delayed thermal annealing times.
