# Supplementary material for: Reducing Defects in Organic-Lead Halide Perovskite Film by Delayed Thermal Annealing Combined with KI/I2 for Efficient Perovskite Solar Cells
Source: Nanomaterials (Basel). 2021 Jun 18;11(6):1607. doi: 10.3390/nano11061607 (PMC8234527; doi:10.3390/nano11061607)
Supplement: Supplementary file 1 [file nanomaterials-11-01607-s001.zip › nanomaterials-1235447-supplementary.pdf]

nanomaterials

MDPI

Supplementary information

Reducing Defects in Organic-Lead Halide Perovskite Film by Delayed Thermal Annealing Combined with KI/I_2 for Efficient Perovskite Solar Cells

Kun-Mu Lee [1,2,3,4,*], Shun-Hsiang Chan [1,3], Wei-Hao Chiu [3], Seoungjun Ahn [1], Chang-Chieh Ting [1], Yin-Hsuan Chang [1], Vembu Suryanarayanan [5], Ming-Chung Wu [1,2,3,*] and Ching-Yuan Liu [6,*]

1 Department of Chemical and Materials Engineering, Chang Gung University, Taoyuan 33302, Taiwan; shunhsiangchan@gmail.com (S.-H.C.); jun864@naver.com (S.A.); kero5206@gmail.com (C.-C.T.); cgu.yinhsuanchang@gmail.com (Y.-H.C.)
2 Division of Neonatology, Department of Pediatrics, Chang Gung Memorial Hospital, Linkou, Taoyuan 33305, Taiwan
3 Green Technology Research Center, Chang Gung University, Taoyuan 33302, Taiwan; weihoa.chiu@gmail.com
4 Center for Reliability Sciences and Technologies, Chang Gung University, Taoyuan 33302, Taiwan
5 Electroorganic and Materials Electrochemistry Division, CSIR-Central Electrochemical Research Institute, Karaikudi 630003, India; vidhyasur@yahoo.co.in
6 Department of Chemical and Materials Engineering, National Central University, Jhongli District, Taoyuan 32001, Taiwan
* Correspondence: kmlee@mail.cgu.edu.tw (K.-M.L.); mingchungwu@cgu.edu.tw (M.-C.W.); cyliu0312@ncu.edu.tw (C.-Y.L.)

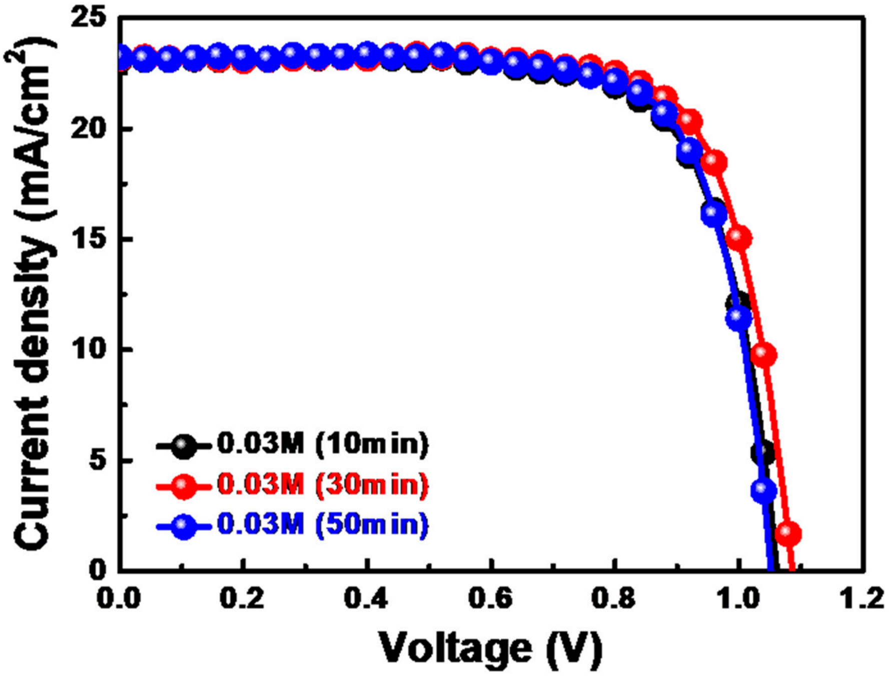

Figure S1. The J-V curves of PSCs with 30 mM KI under various thermal baking time.

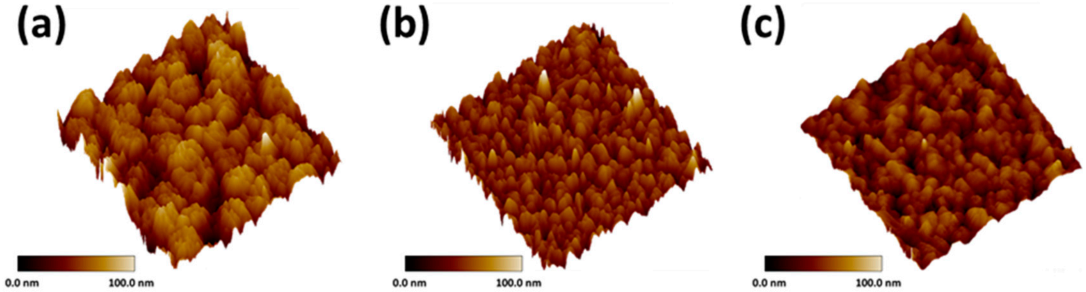

Figure S2. The AFM images of perovskite film with 30 mM KI at different delay-annealing times. (**a**) 0 min, (**b**) 30 min and (**c**) 60 min.

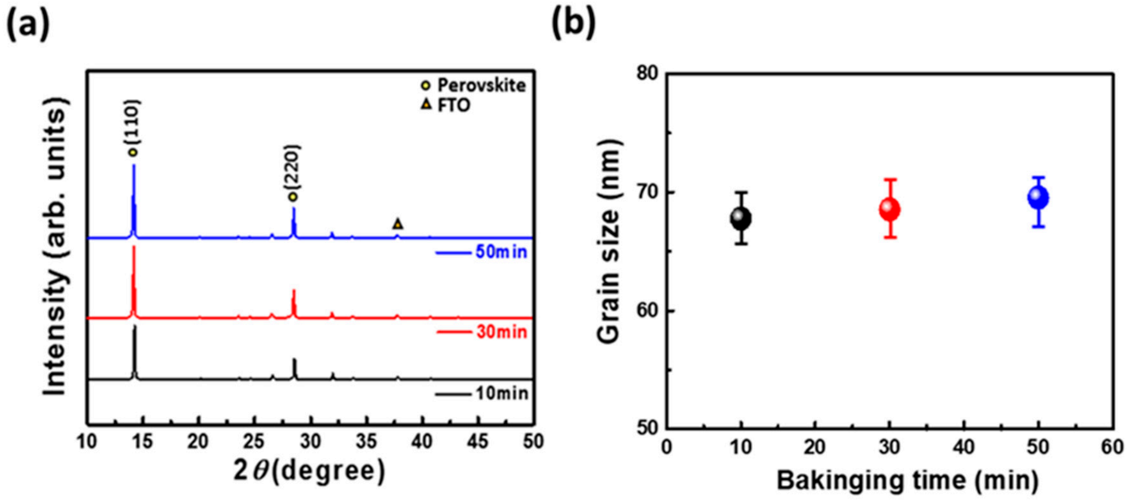

Figure S3. (**a**) XRD analysis of perovskite films with 0.03M KI under various thermal baking times. (**b**) Crystal size in perovskite films with 0.03M KI under various thermal baking times.

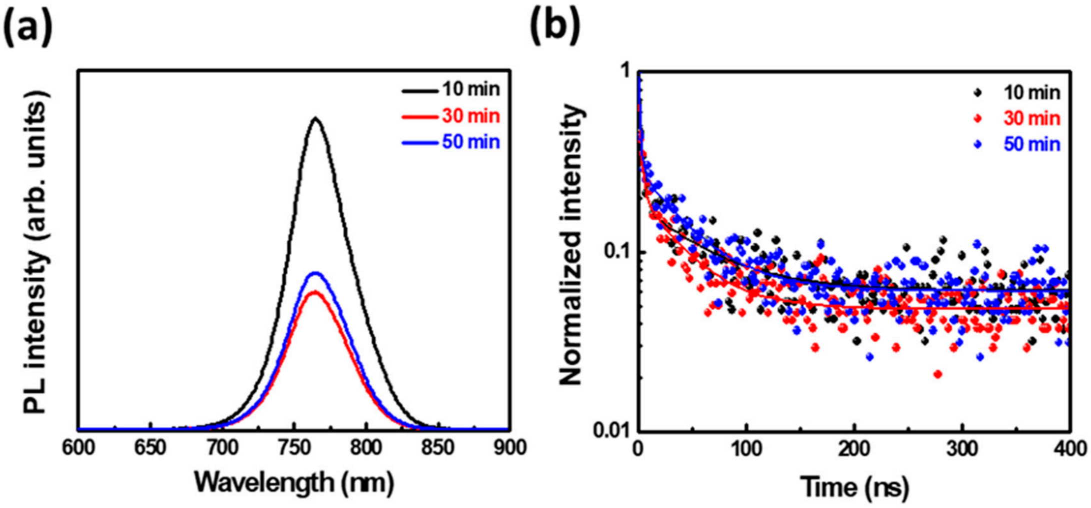

Figure S4. (**a**) PL and (**b**) TRPL of perovskite films with 0.03M KI on TiO_2/FTO glass under various thermal baking times.

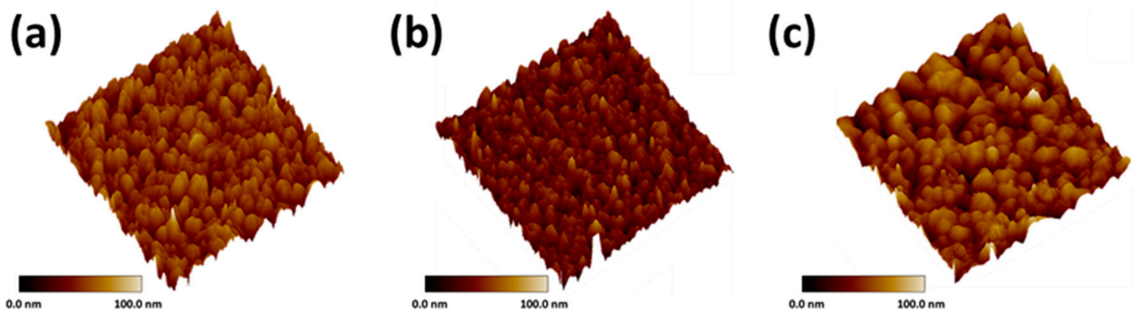

Figure S5. AFM of perovskite films with 0.03M KI/I_2 on TiO_2/FTO glass at different delayed annealing times. (**a**) 10 min, (**b**) 30 min and (**c**) 60 min.

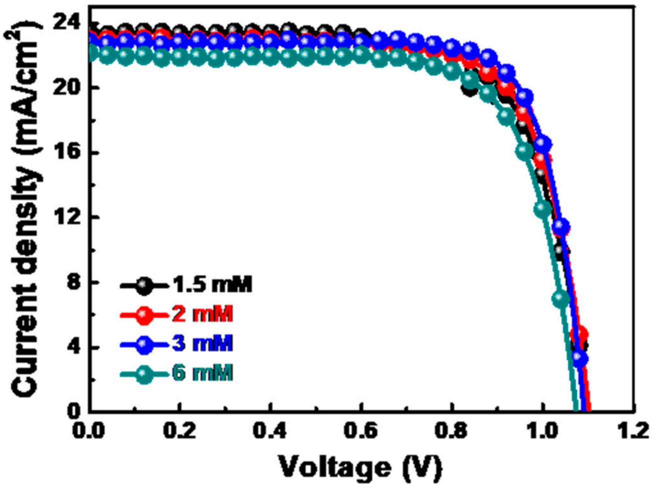

Figure S6. The J-V curves of PSCs with 30 mM KI and various concentrations of I_2.

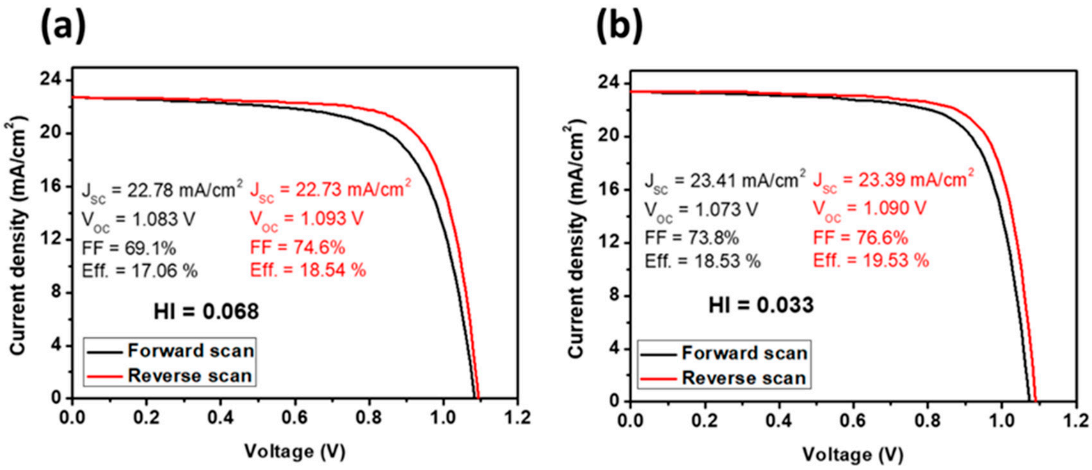

Figure S7. The J-V curves of PSCs by forward (Black) and reverse scan (Red). (**a**) Pristine and (**b**) KI (30 mM) + I_2 (3 mM).

Table S1. The R_{mas} and RMS of perovskite films with 30 mM KI prepared at different delayed annealing times.

Time (min)	0	30	60
R_{max} (nm)	482	429	154
RMS (nm)	53	36	19

Table S2. Photovoltaic characteristics of PSCs with 30 mM KI at different thermal baking times.

Time (min)		V_{OC} (V)	J_{SC} (mA/cm^2)	FF (%)	PCE (%)
10	Maximum	1.061	23.09	73.8	18.10
	Mean deviation	1.060 ± 0.014	22.65 ± 0.41	71.6 ± 1.4	17.22 ± 0.58
30	Maximum	1.086	23.19	75.1	18.93
	Mean deviation	1.069 ± 0.014	22.73 ± 0.30	75.5 ± 1.9	18.21 ± 0.39
50	Maximum	1.052	23.22	74.7	18.26
	Mean deviation	1.061 ± 0.010	22.88 ± 0.29	73.2 ± 1.4	17.77 ± 0.42

Table S3. The carrier lifetimes of perovskite with 30 mM KI fitting from TRPL analysis at different thermal baking times.

Thermal Baking Time (min)	A_1 (%)	τ_1 (ns)	A_2 (%)	τ_2 (ns)	τ_{avg} (ns)
10	86	2.7	14	58.7	10.5
30	79	2.3	21	39.8	10.2
50	73	1.9	27	44.1	13.3

Table S4. R_{max} and RMS of perovskite films with KI (30 mM)/I_2 (3 mM) at various delayed thermal annealing times.

Time (min)	0	30	60
R_{max} (nm)	366	366	225
RMS (nm)	34	28	26
